# Supplementary material for: RGS9‐2 rescues dopamine D2 receptor levels and signaling in DYT1 dystonia mouse models
Source: EMBO Mol Med. 2018 Dec 14;11(1):e9283. doi: 10.15252/emmm.201809283 (PMC6328939; doi:10.15252/emmm.201809283)
Supplement: Supplementary file 1 — Appendix [file EMMM-11-e9283-s001.pdf]

Appendix

Table of contents:

Table S1. Primary antibodies used for immunoblotting.

Table S2. Primary antibodies used for confocal imaging.

Appendix Table S1. Primary antibodies used for immunoblotting.

Antibody	Species	Diluition	Supplier/code	References
Anti-p62	Guinea Pig	1:1000	Progen (GP62-C)	https://www.labome.com/product/Progen/gp62-c.html<br>Waguri and Komatsu *Methods Enzymol.* 2009
Anti-LC3	Mouse	1:250	NanoTools (0231-100)	Lin et al., *Cell Death Disease* 2014
Anti-RGS9	Goat	1:1000	Santa Cruz (sc-8142)	Mancuso et al., *J Neurochem 2010*<br>Napolitano et al., *Neurobiol. Dis.* 2010
Anti-D2R	Rabbit	1:1000	Millipore (AB5084P)	Stojanovic et al., *Amino Acids* 2017
Anti-TorsinA	Rabbit	1:800	Abcam (AB34540)	Puglisi et al., *PlosOne* 2013
Anti-RGS7	Goat	1:200	Santa Cruz (sc-8139)	Lopez-Fando et al., *Neuropsychopharmacol* 2005<br>Terzi et al., *Neuropsychopharmacol* 2012
Anti-Gβ5	Rabbit	1:1000	Millipore (071208)	Terzi et al., *Neuropsychopharmacol* 2012
Anti-R7BP	Goat	1:500	Santa Cruz (sc-50170)	Terzi et al., *Neuropsychopharmacol* 2012
Anti-Rab4	Rabbit	1:1000	Abcam (ab13252)	Peng et al., *JBC* 2012
Anti-spinophilin / neurabin-II	Rabbit	1:3000	Sigma Aldrich (5162)	Feng et al., *Proc Natl Acad Sci U S A.* 2000
Anti-LAMP2	Rat	1:200	Genetex (GTX13524)	Rothaug et al., *Acta Neuropathol Comm* 2015
Anti-β arrestin-2	Mouse	1:500	Santa Cruz (sc-13140)	Beaulieu et al., *Cell* 2005<br>Lee et al., *J Neurosci* 2011
Anti-GFP	Rabbit	1:200	Santa Cruz (sc-8334)	Kretz et al.,*Molecular Therapy* 2003

Appendix Table S2. Primary antibodies used for confocal imaging.

Antibody	Species	Diluition	Supplier/code	References
Anti-RGS9	Goat	1:1000	Santa Cruz (sc-8142)	Mancuso et al., *J Neurochem* 2010
Anti-ChAT	Goat	1:500	Novus Biol. (NBP1-30052)	Ponterio et al., *Neuropharmacol* 2013
Anti-p62	Guinea Pig	1:300	Progen (GP62-C)	Sarkar et al., *Autophagy* 2014
Anti-D2R	Rabbit	1:500	Millipore (AB5084P)	Song et al., *Front Behav Neurosci* 2014<br>Rajput et al., *Neurosci Res* 2009
Anti-PDI	Mouse	1:500	StressGene (SPA891)	O'Farrel et al., *Neurosci* 2009
