# Supplementary material for: RGS9‐2 rescues dopamine D2 receptor levels and signaling in DYT1 dystonia mouse models
Source: EMBO Mol Med. 2018 Dec 14;11(1):e9283. doi: 10.15252/emmm.201809283 (PMC6328939; doi:10.15252/emmm.201809283)
Supplement: Supplementary file 2 — Expanded View Figures PDF [file EMMM-11-e9283-s002.pdf]

Expanded View Figures

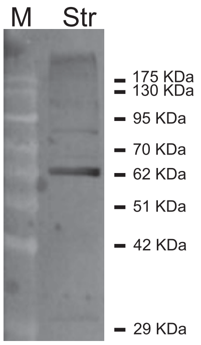

Figure EV1. Representative DRD2 immunoblotting of striatal tissue.
Pattern of DRD2 immunolabeling in the striatum of adult wild-type Tor1a$^{+/+}$ mice. 30 μg of striatal (Str) lysate was loaded. DRD2 antibody recognized a prominent band at ~ 63 kDa.

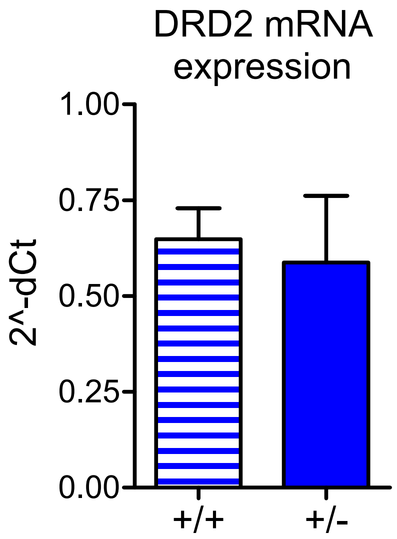

Figure EV2. Similar DRD2 mRNA expression in *Tor1a*$^{+/+}$ and *Tor1a*$^{+/-}$ striatum.
DRD2 mRNA expression is unaltered in the striatum of *Tor1a*$^{+/-}$ mice (N = 3) with respect to wild-types (N = 3), as determined by qRT–PCR (Mann–Whitney test $P > 0.05$). Data are represented as mean ± SEM.

© 2018 The Authors
