# Supplementary material for: RGS9‐2 rescues dopamine D2 receptor levels and signaling in DYT1 dystonia mouse models
Source: EMBO Mol Med. 2018 Dec 14;11(1):e9283. doi: 10.15252/emmm.201809283 (PMC6328939; doi:10.15252/emmm.201809283)

Figure 1A1 -torsinA

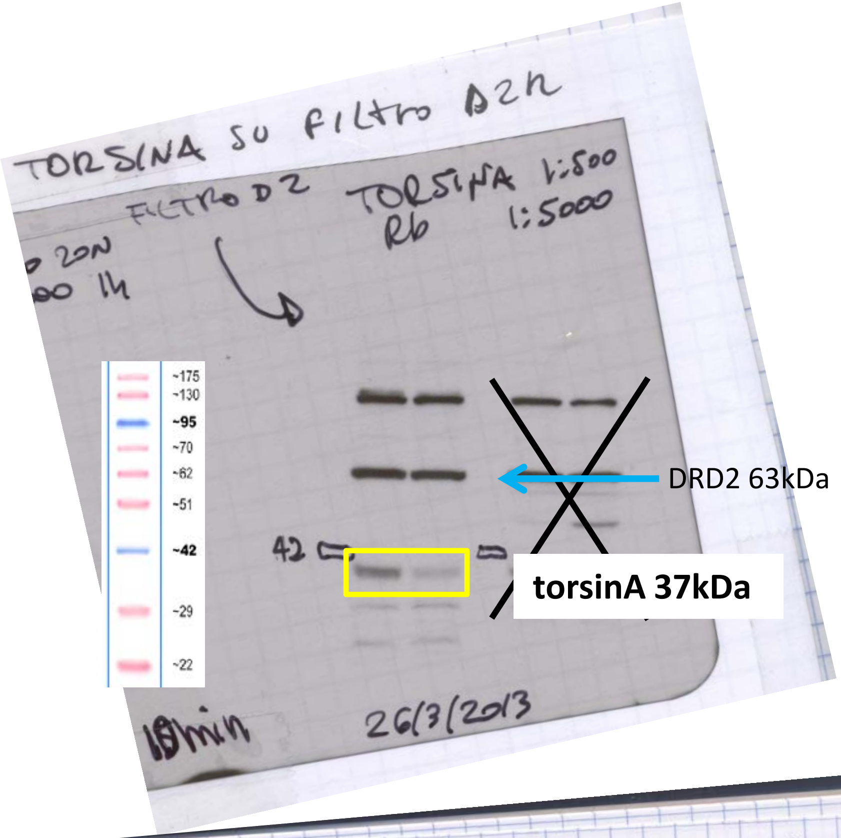

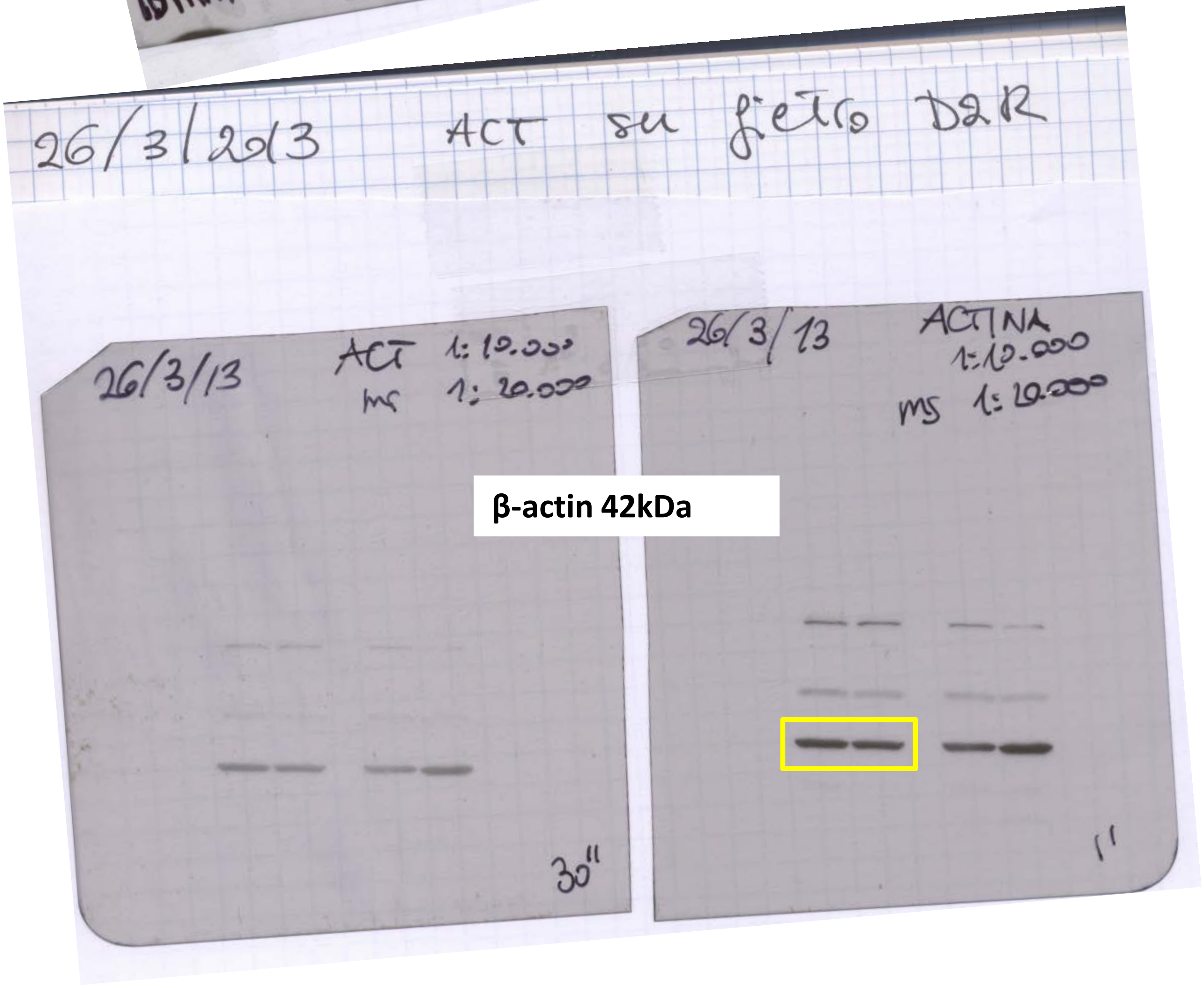

Figure 1A1 – RGS7

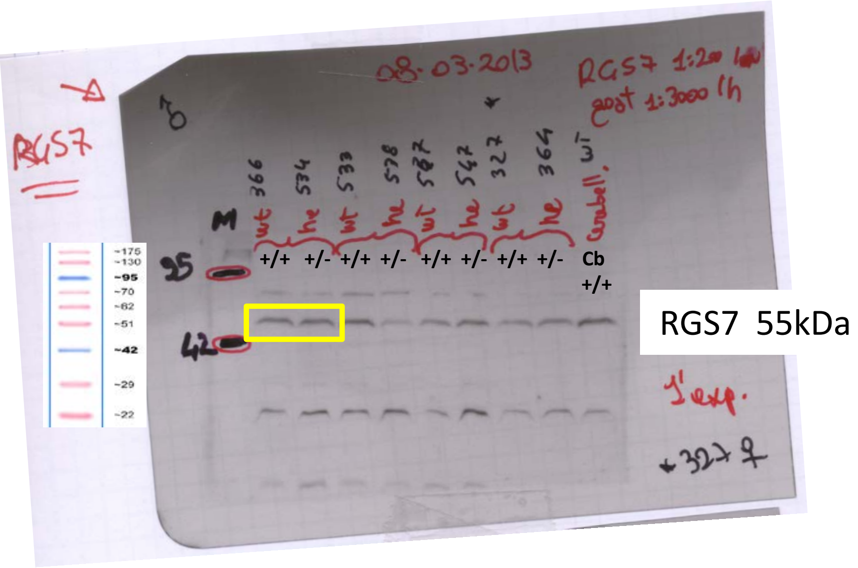

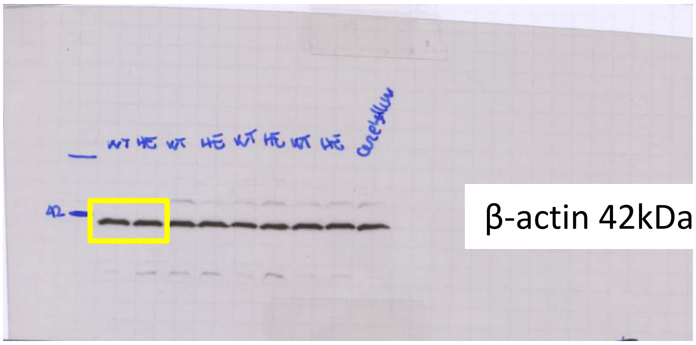

Figure 1A1 – RGS9-2, DRD2

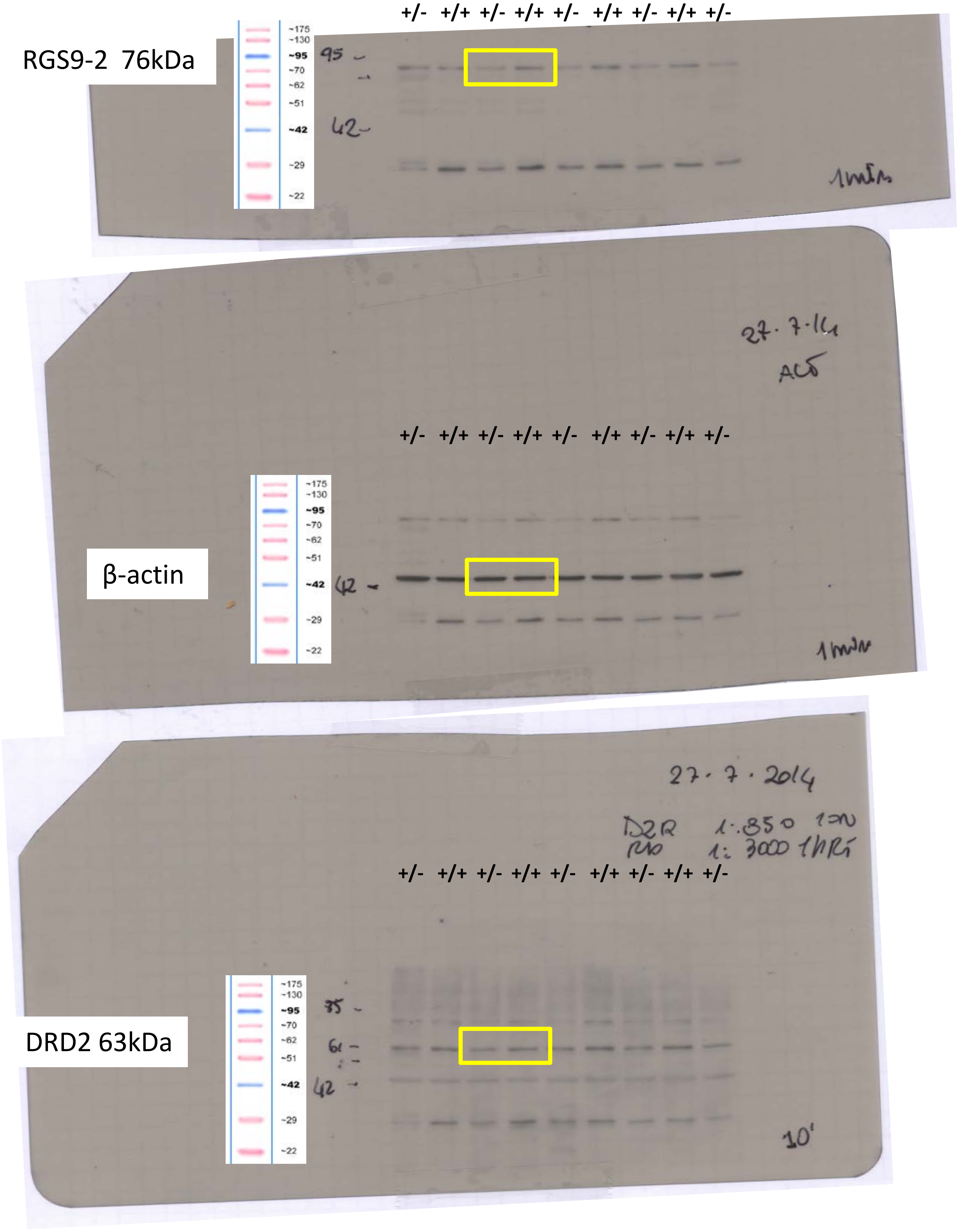

Figure 1A2

Tor1a+/+ M Tor1a+/-
P7 P14 P21 P60 P7 P14 P21 P60 P14
k20 k10 k14 2 52 k23 k9 k12 171 k9

TorsinA 37kDa

β-actin 42kDa

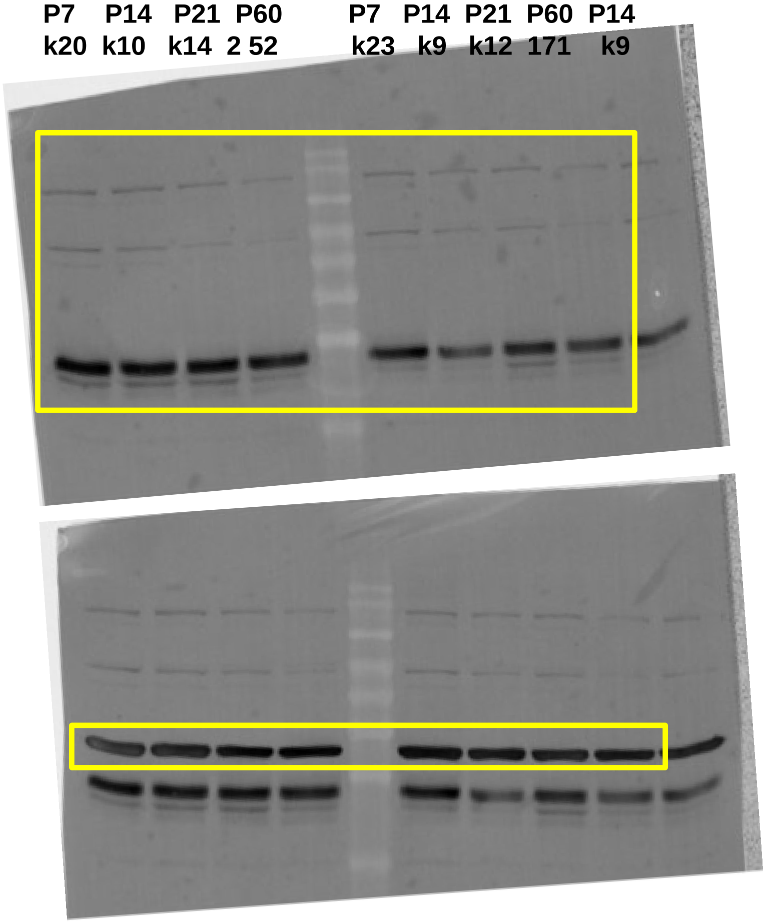

Figure 1A3

Tor1a+/+ M Tor1a+/- M
P7 P14 P21 P60 P7 P14 P21 P60
k26 k32 k17 385 k25 k30 k18 372

DRD2 63kDa

β-actin 42kDa

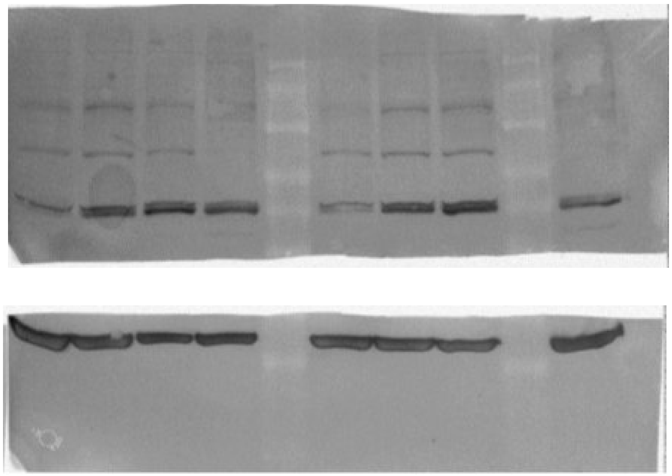

Figure 1A4

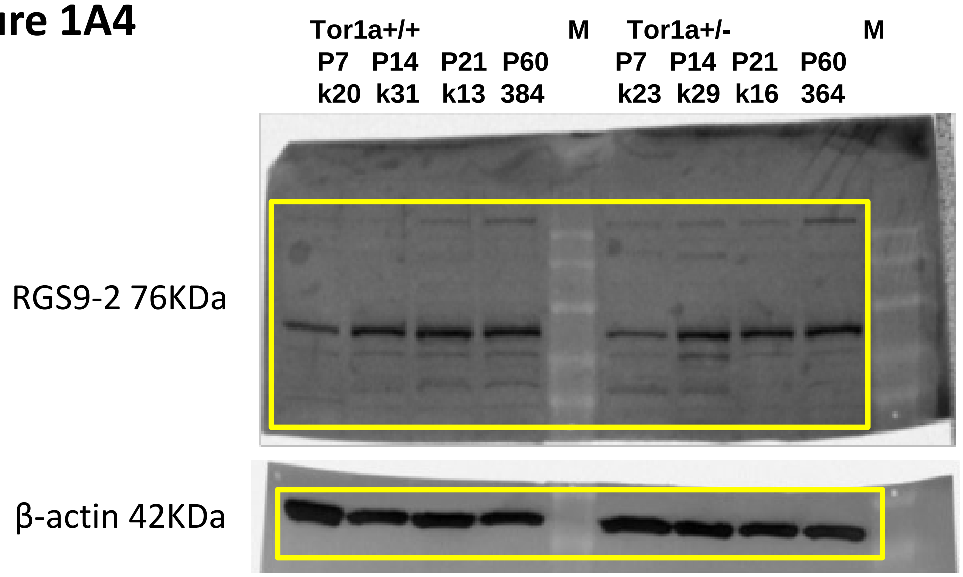

Tor1a+/+
M
Tor1a+/-
M
P7 P14 P21 P60
k20 k31 k13 384
P7 P14 P21 P60
k23 k29 k16 364
RGS9-2 76KDa
β-actin 42KDa

Figure 1B- DRD2,torsinA

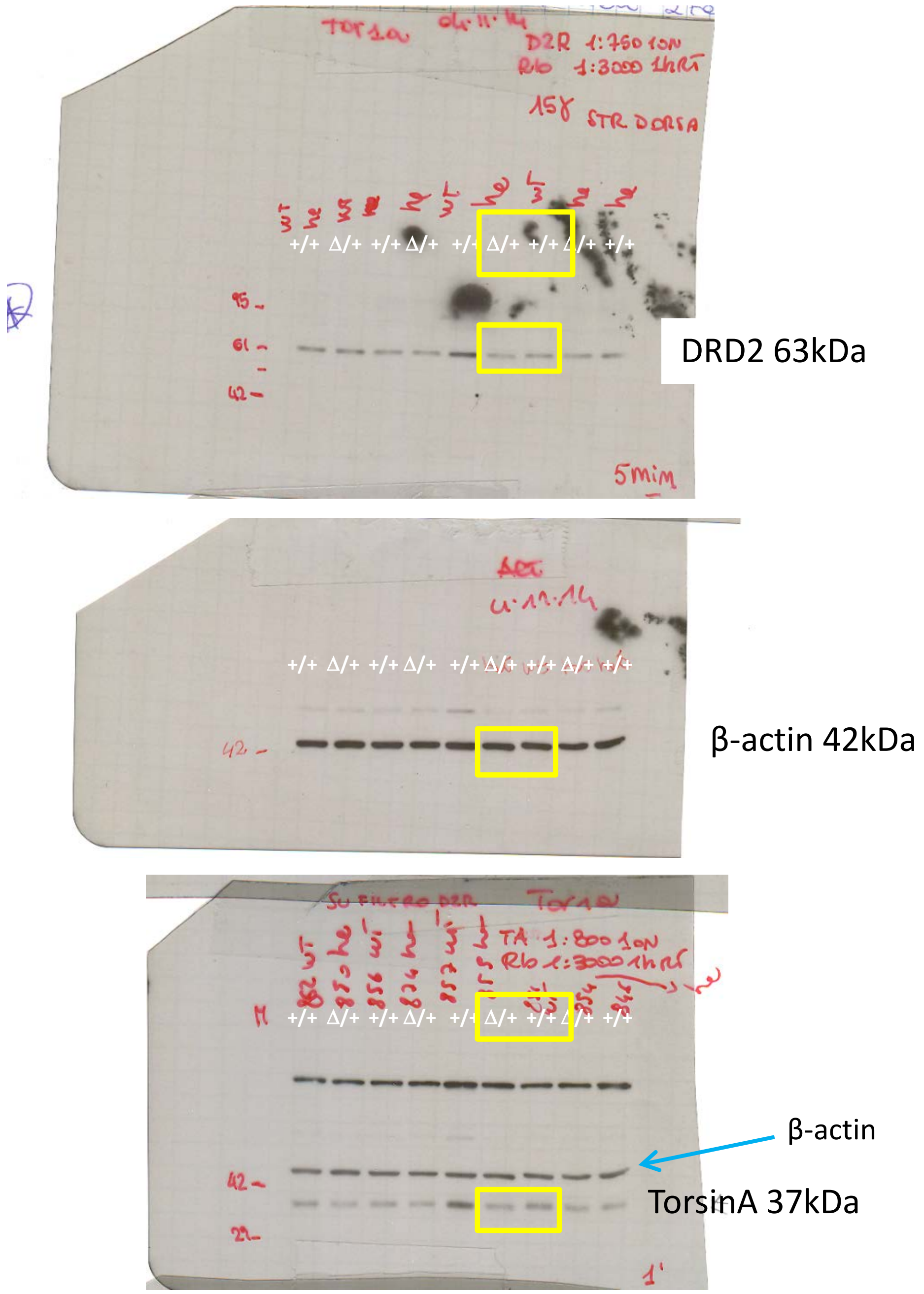

Figure 1B - RGS9-2

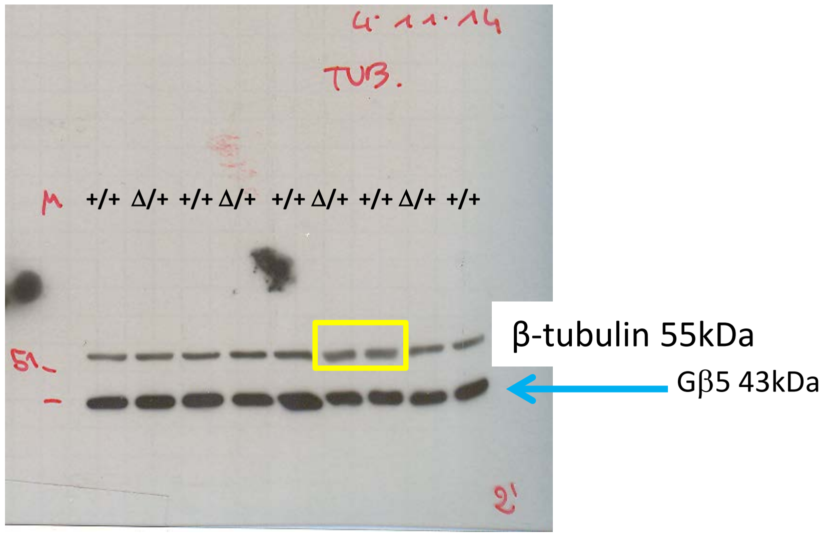

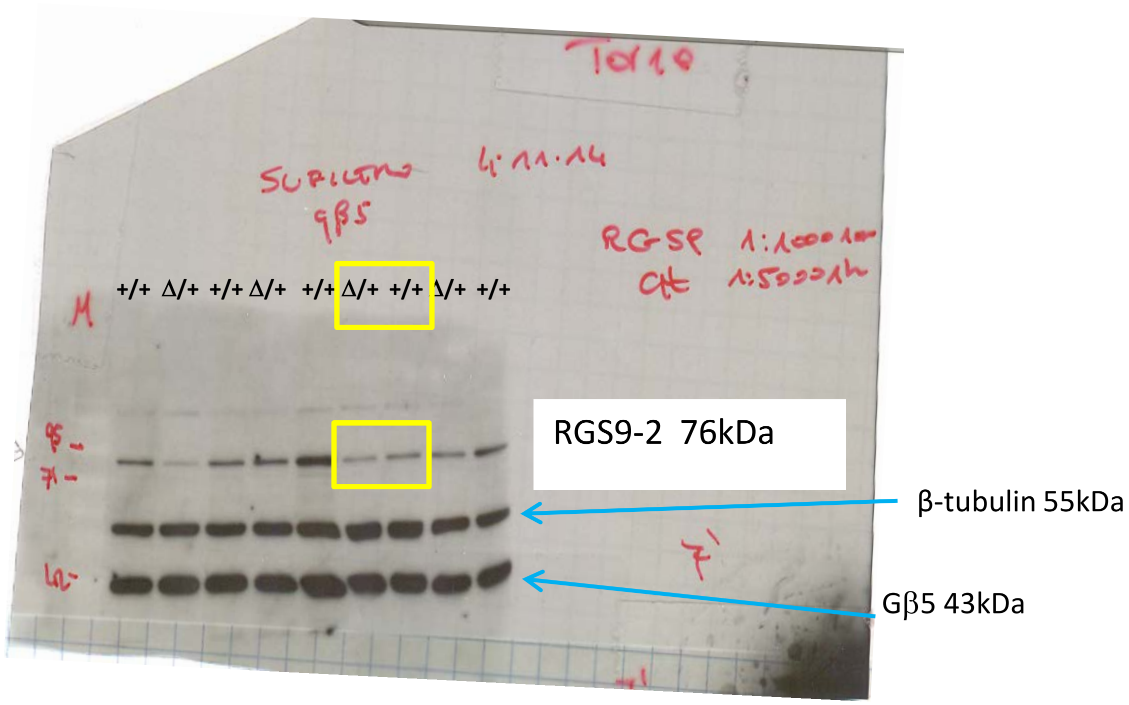

Supplement: Supplementary file 4 — Source Data for Figure 1 [file EMMM-11-e9283-s003.pdf]
