# Supplementary figures and images for: RGS9‐2 rescues dopamine D2 receptor levels and signaling in DYT1 dystonia mouse models
Source: EMBO Mol Med. 2018 Dec 14;11(1):e9283. doi: 10.15252/emmm.201809283 (PMC6328939; doi:10.15252/emmm.201809283)

Figure 2A1- RGS9-2

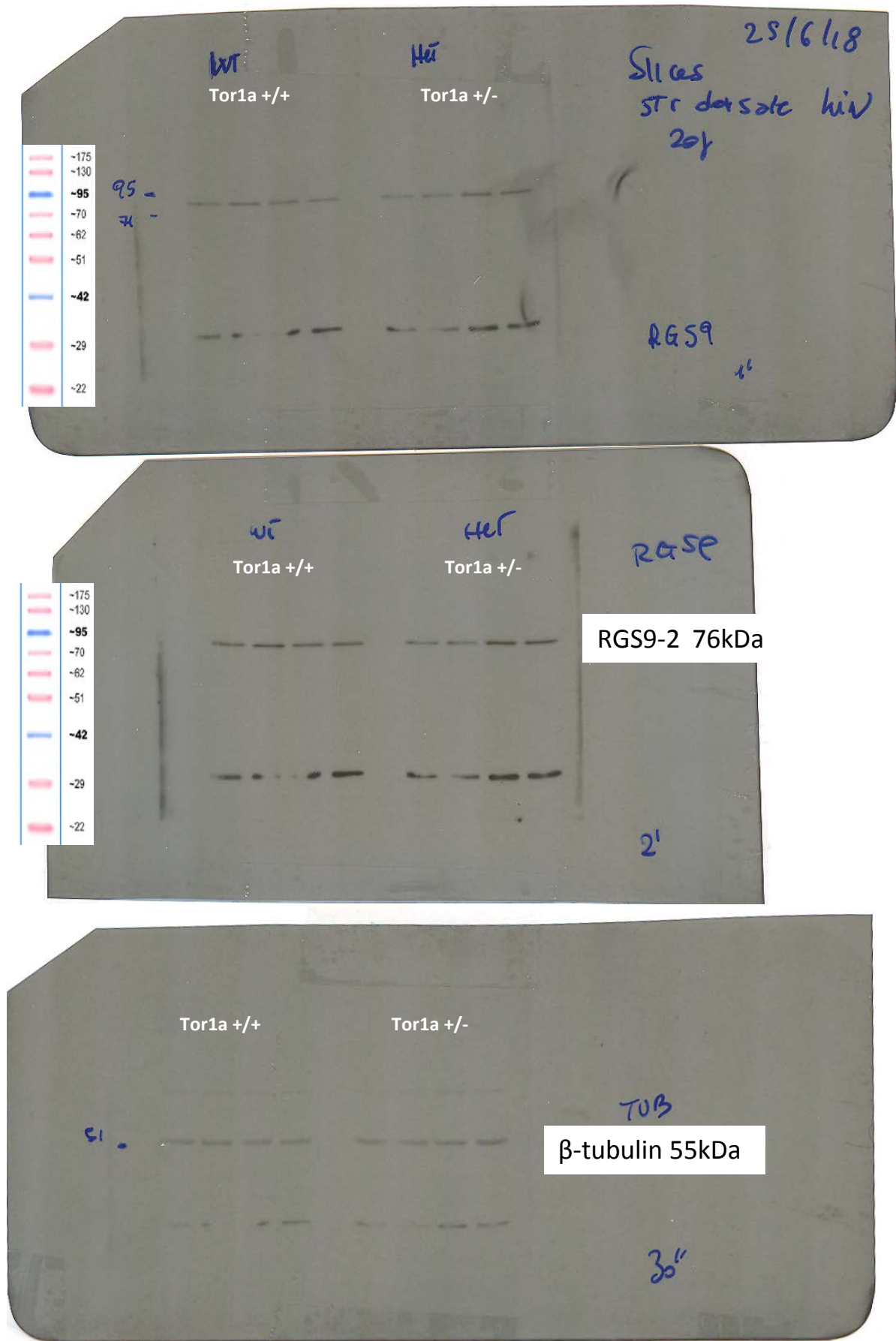

Figure 2A1- R7BP

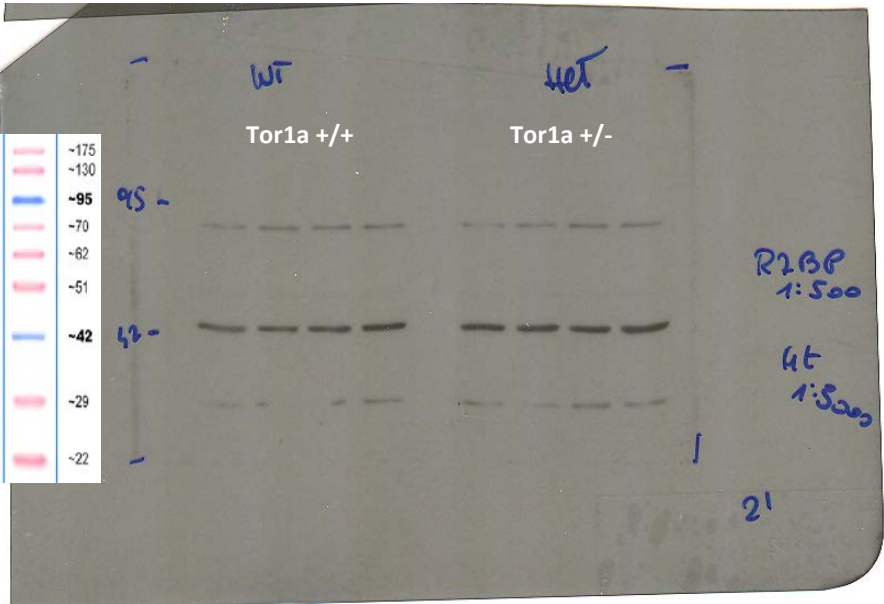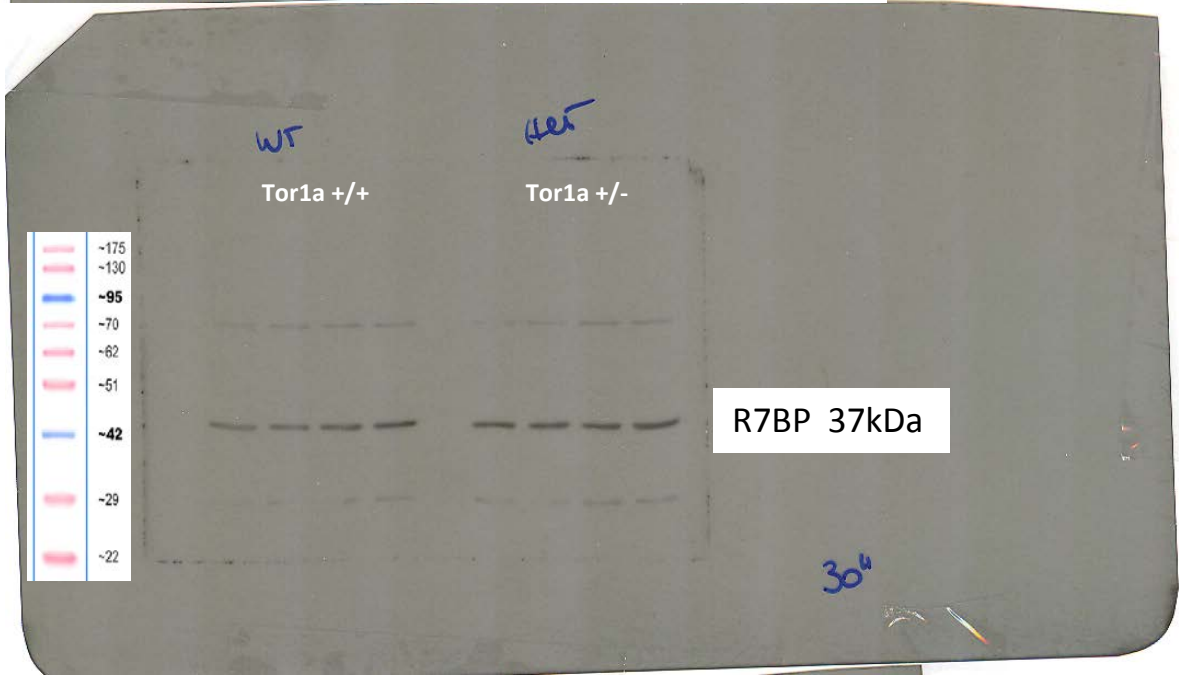

Figure 2A1- Gβ5

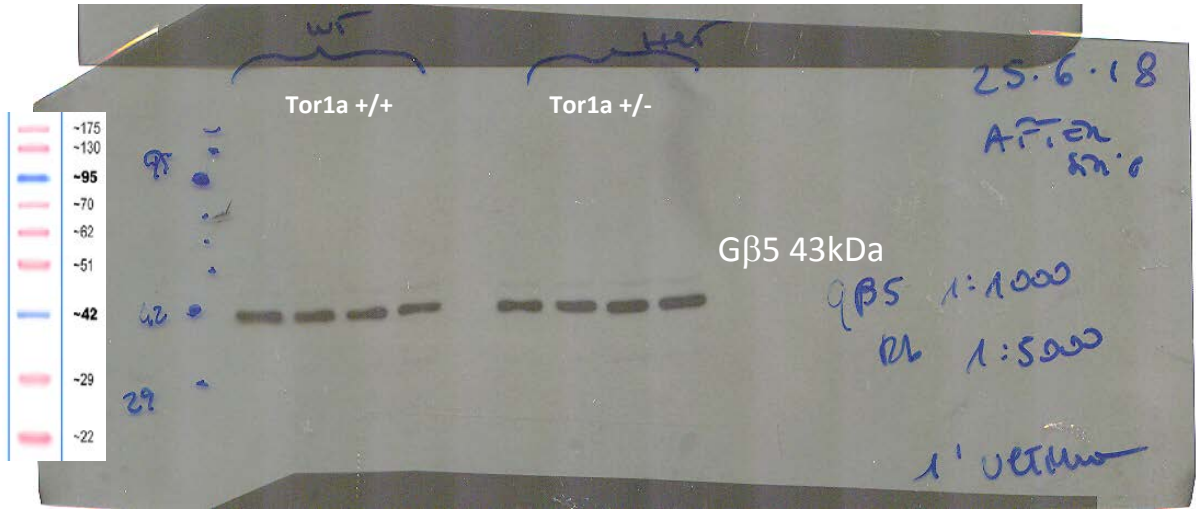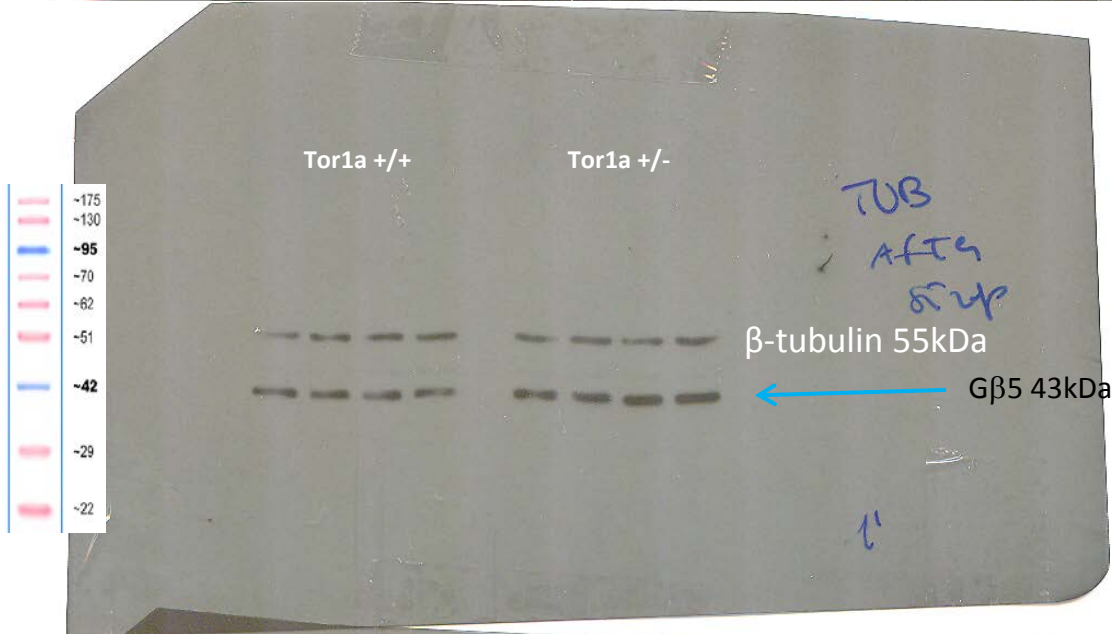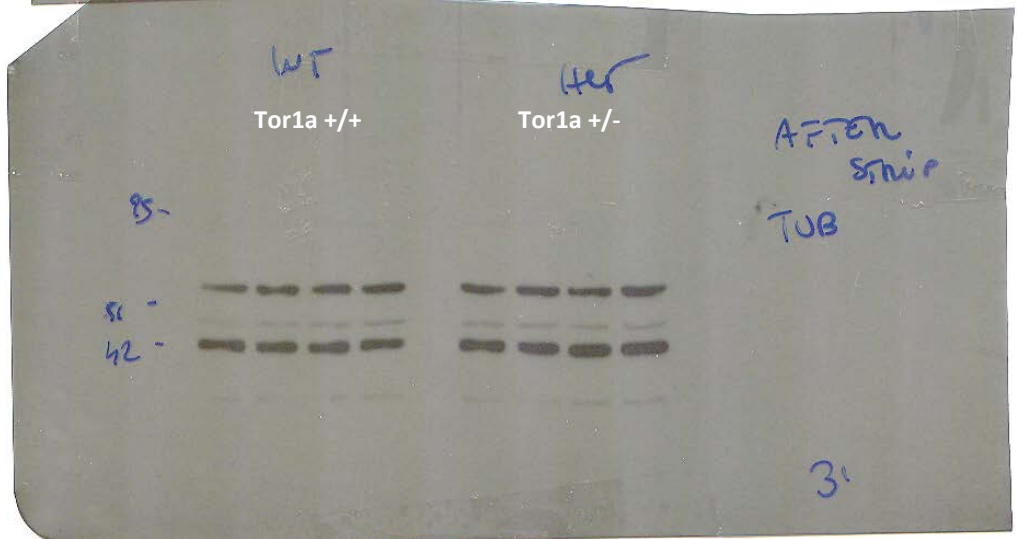

Figure 2B1

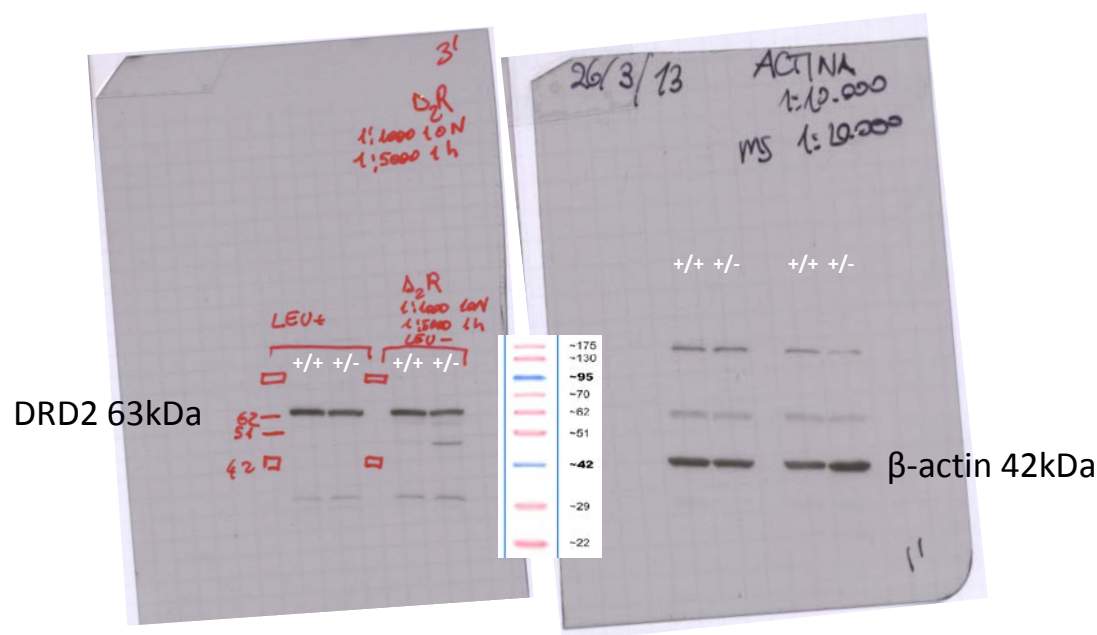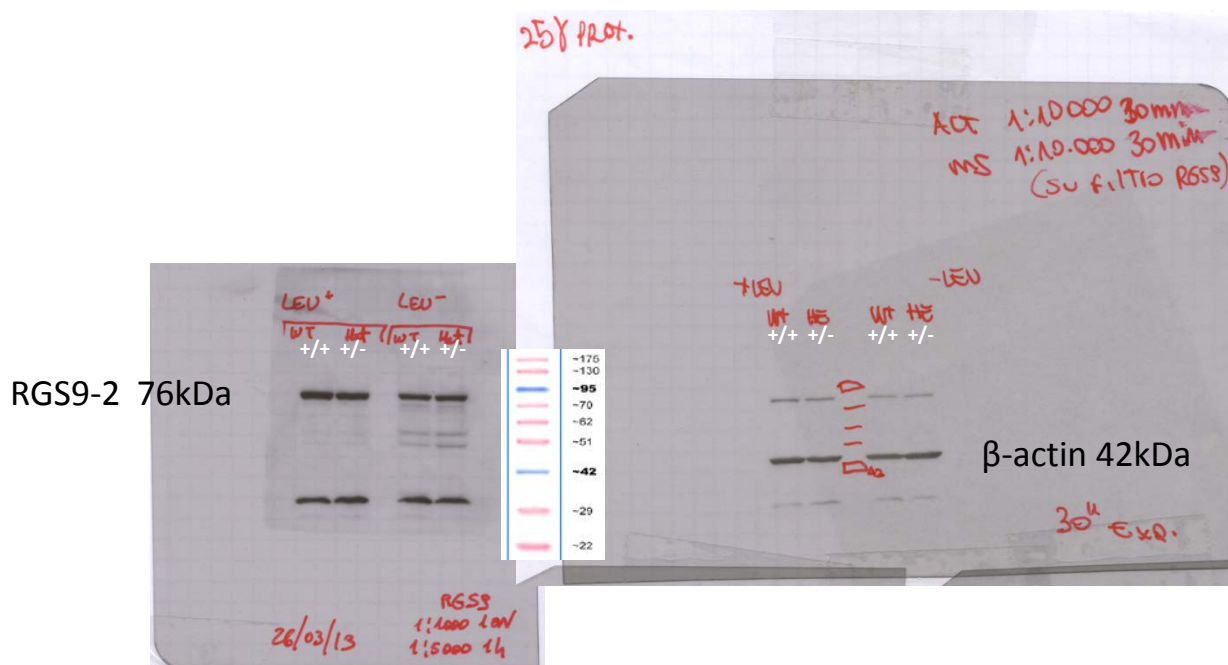

Supplement: Supplementary file 5 — Source Data for Figure 2 [file EMMM-11-e9283-s004.pdf]

### Figure 3A

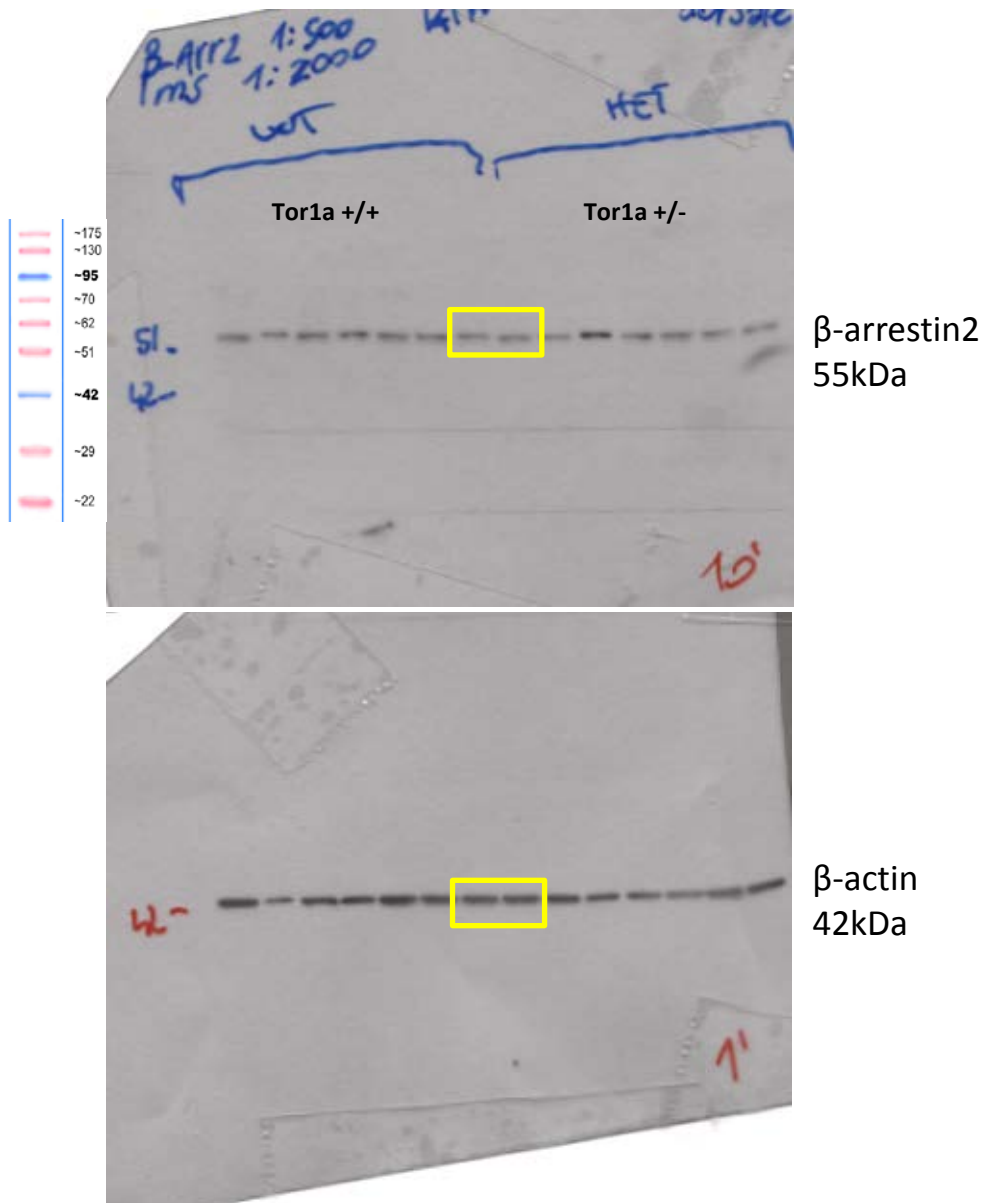

Figure 3B

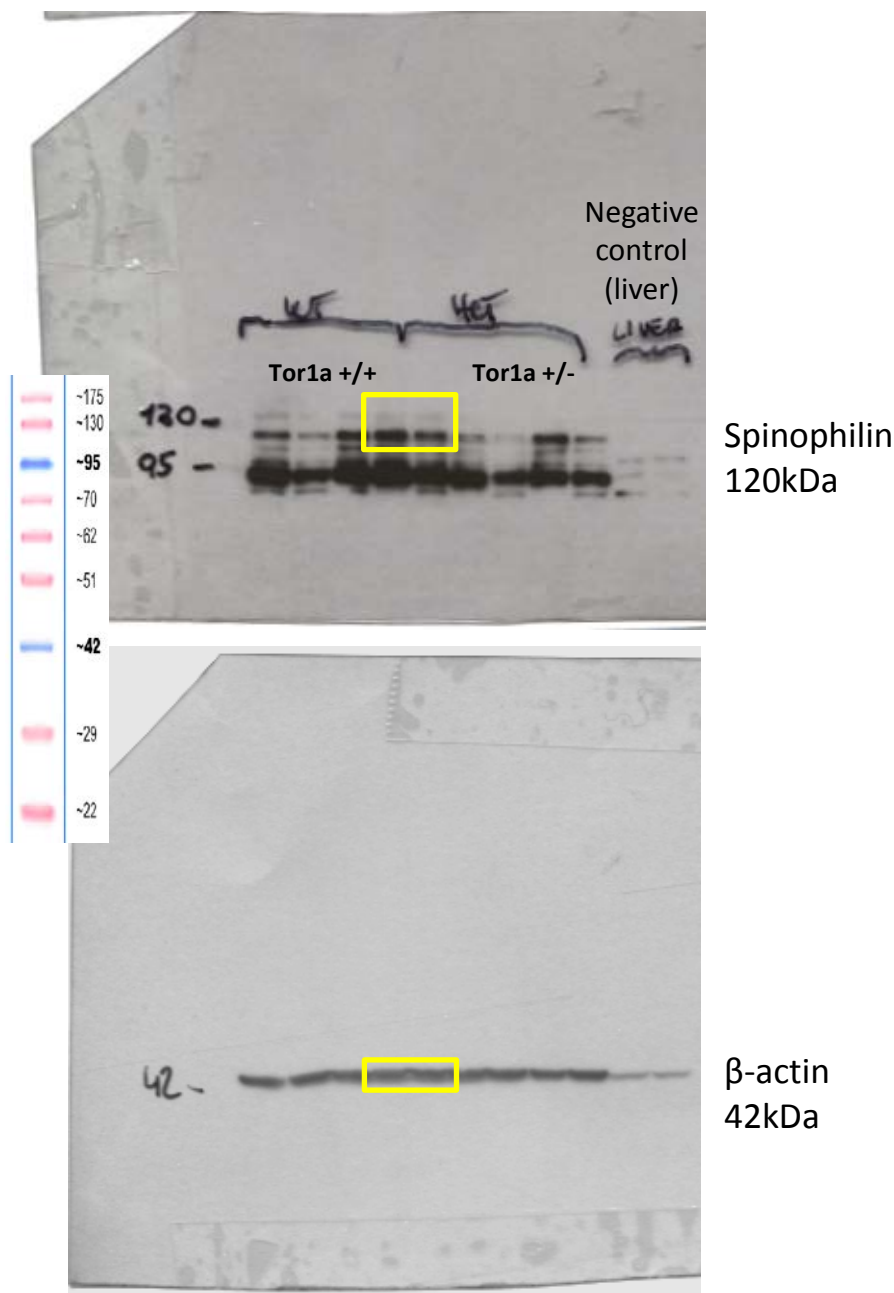

Figure 3C

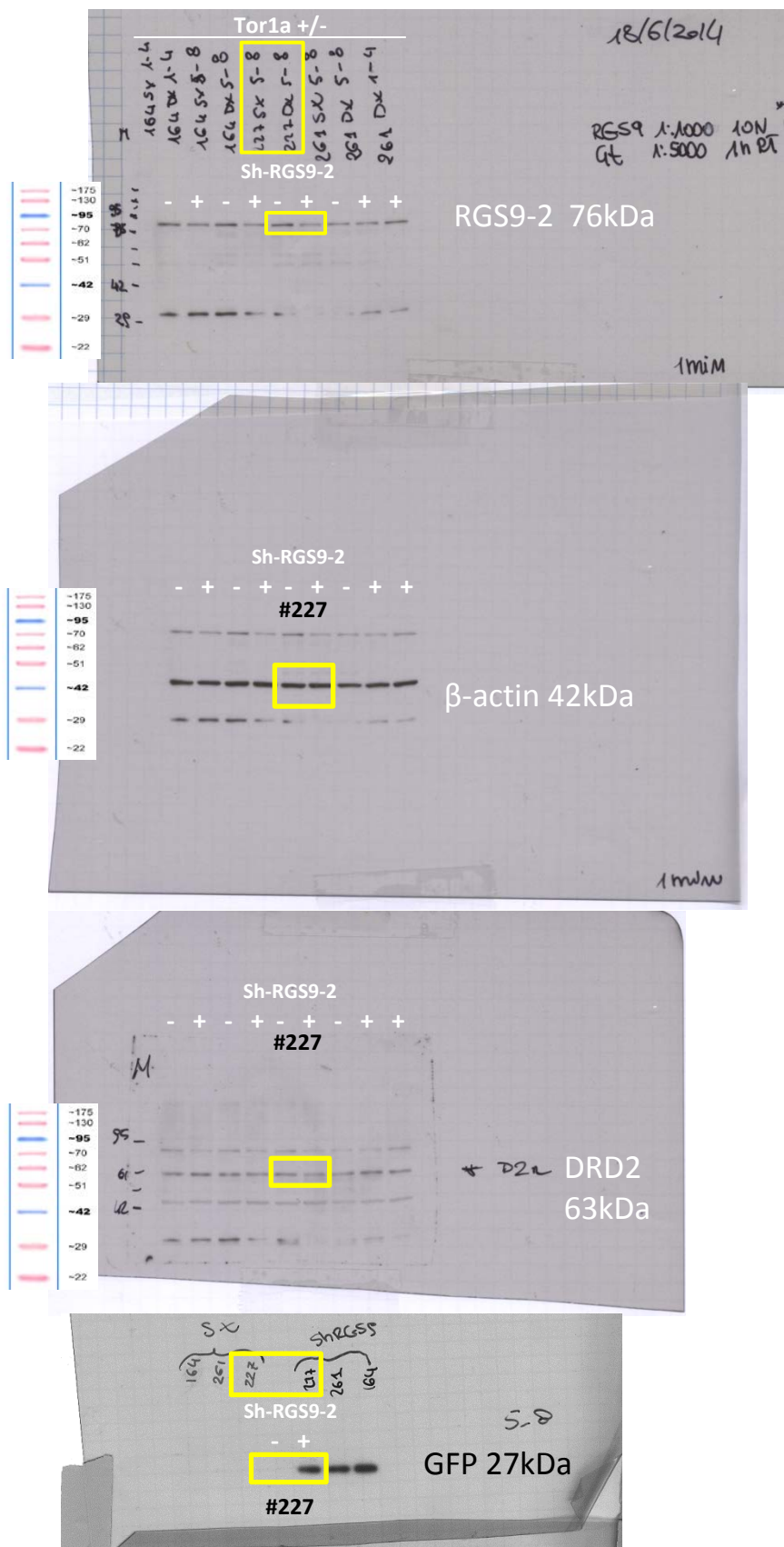

Figure 3D

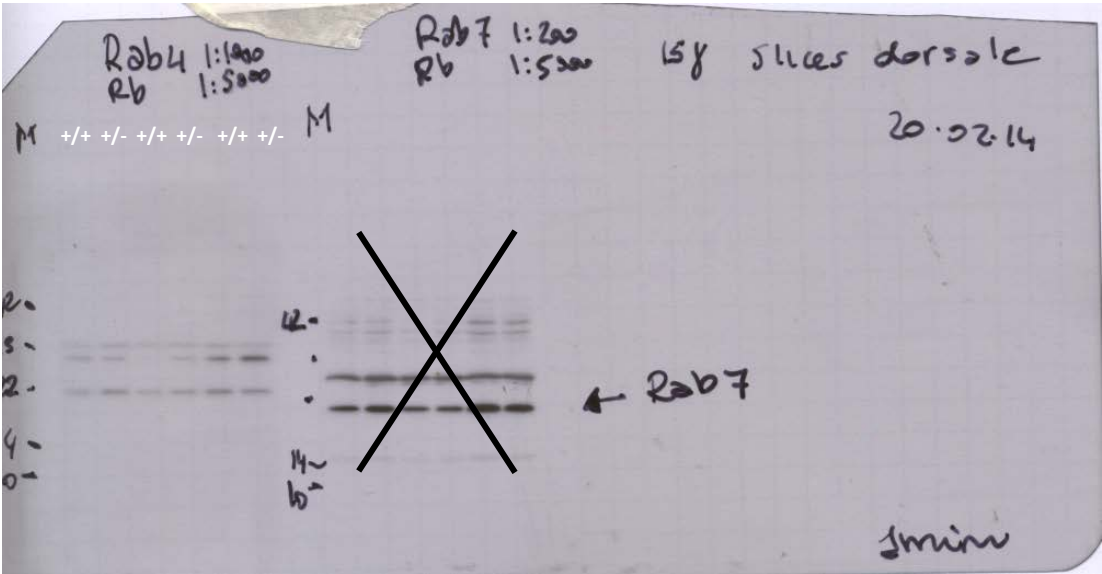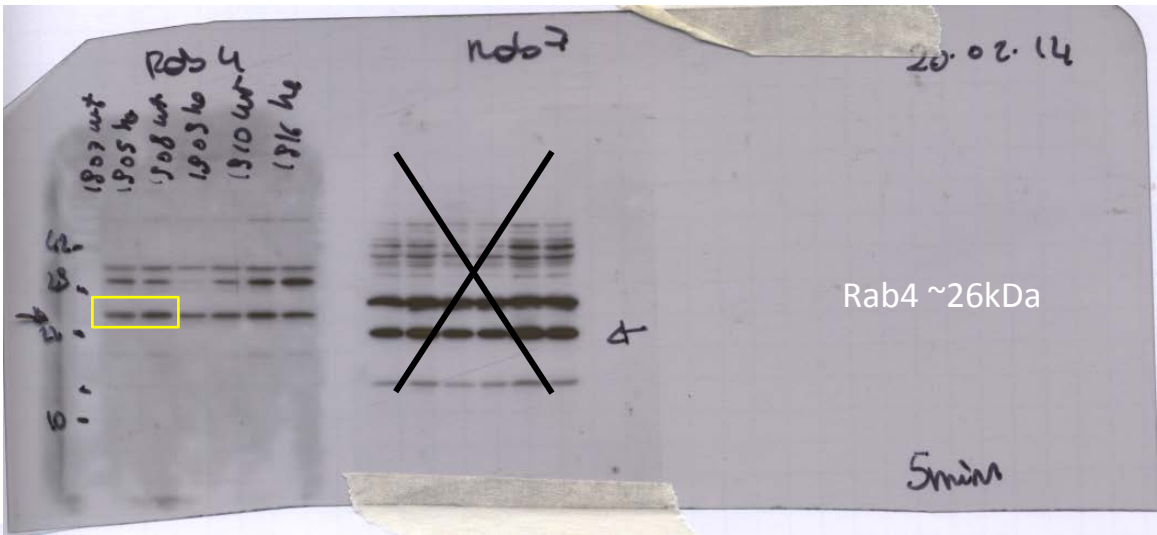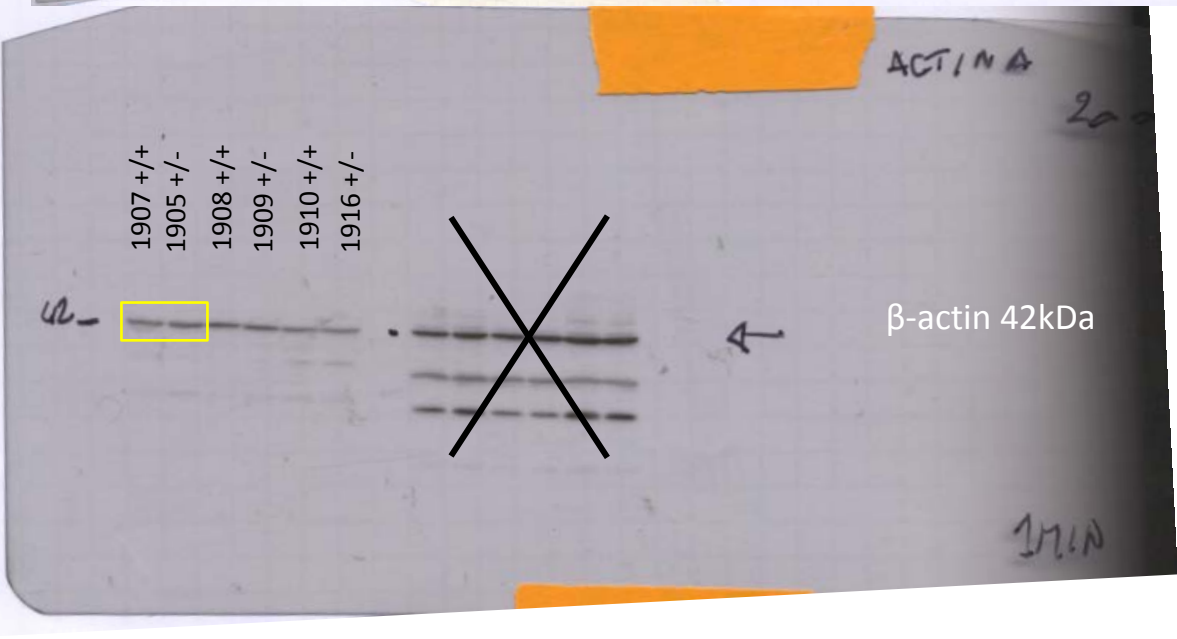

Figure 3G

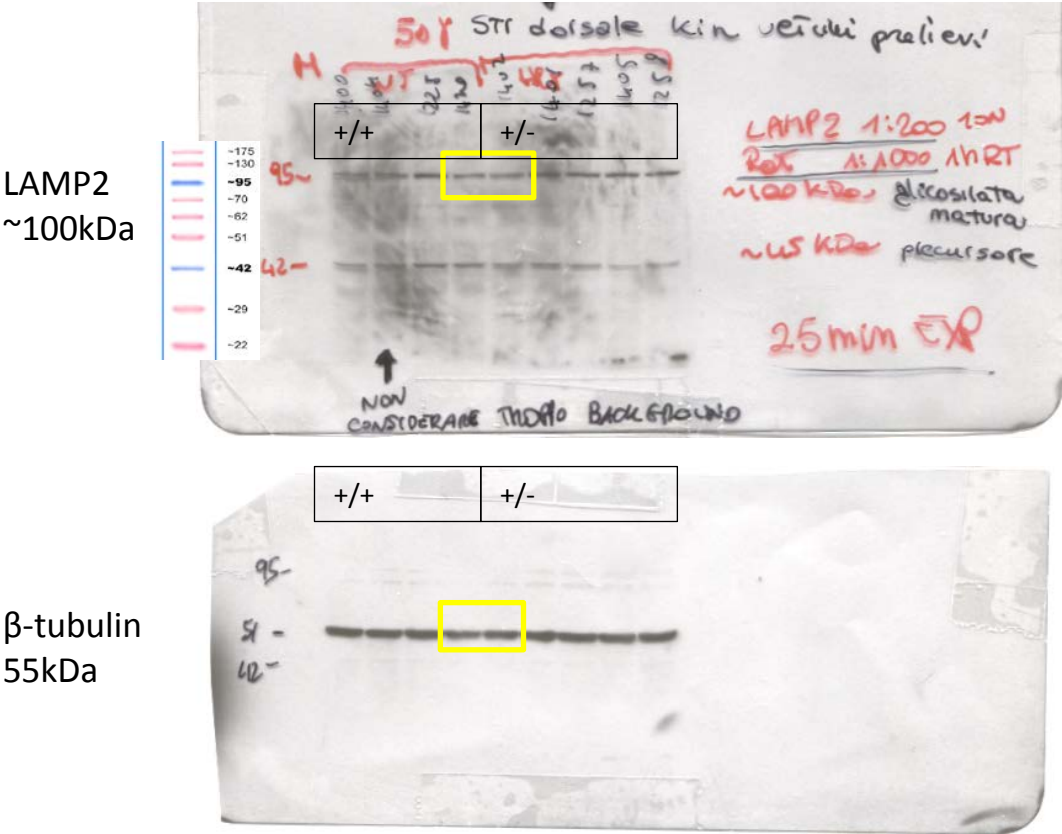

Supplement: Supplementary file 6 — Source Data for Figure 3 [file EMMM-11-e9283-s005.pdf]

Figure 5A

Tor1a<sup>+/+</sup>

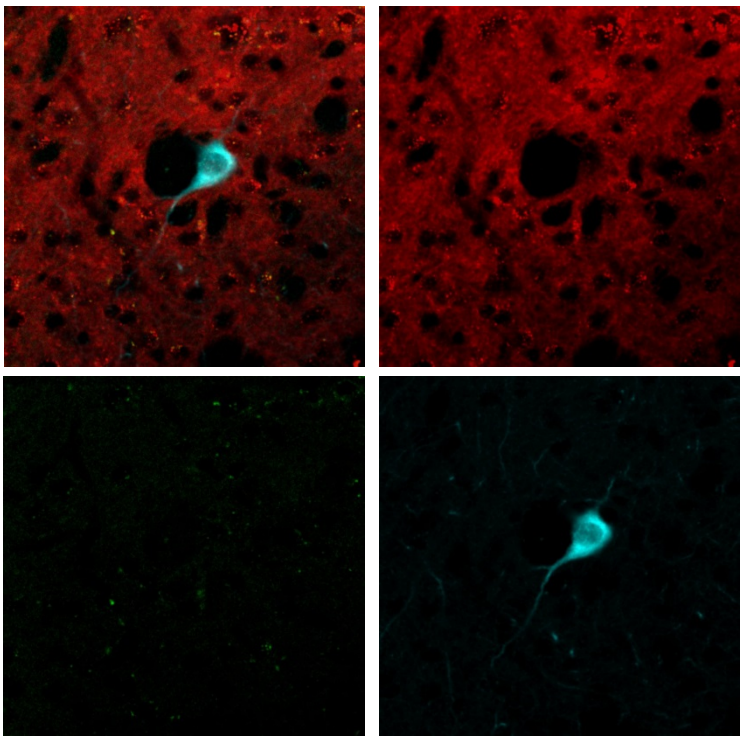

Tor1a<sup>+/-</sup>

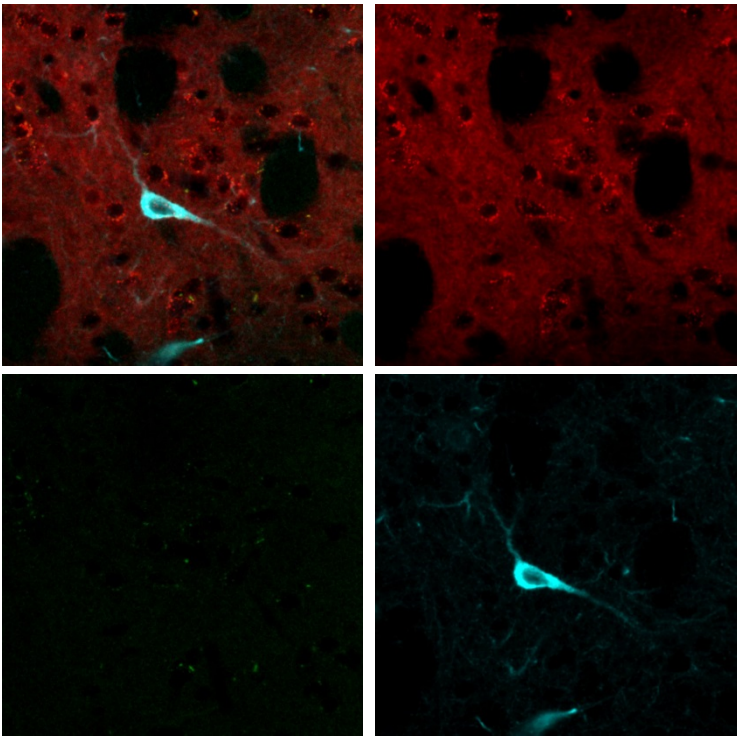

Figure 5C

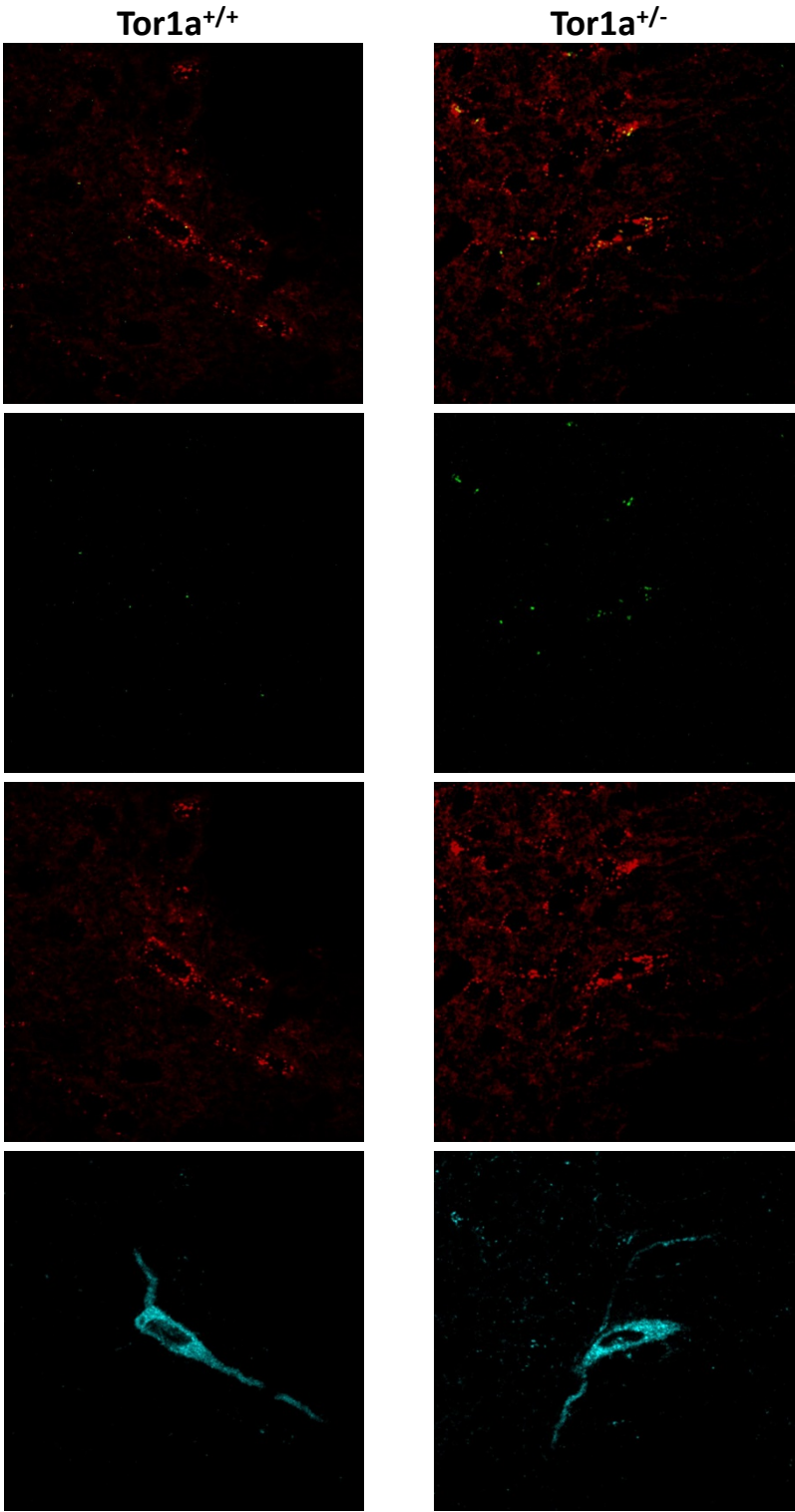

Supplement: Supplementary file 7 — Source Data for Figure 5 [file EMMM-11-e9283-s006.pdf]

[illegible]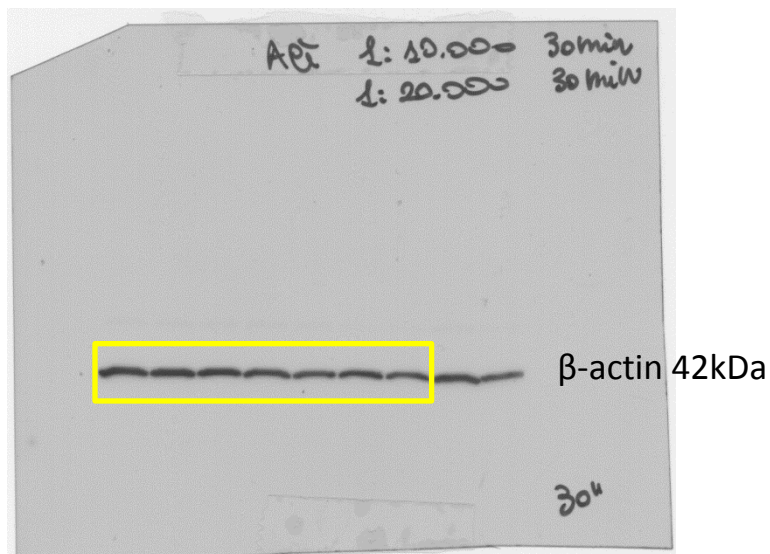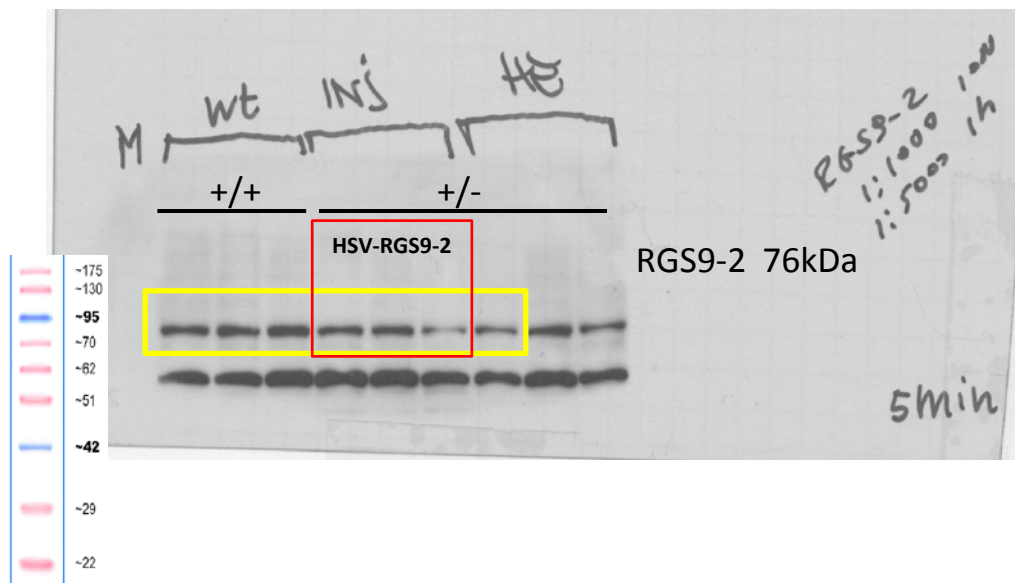

Supplement: Supplementary file 8 — Source Data for Figure 7 [file EMMM-11-e9283-s007.pdf]
